# High incidence of AZF duplications in clan-structured Iranian populations detected through Y chromosome sequencing read depth analysis

**DOI:** 10.1038/s41598-023-39069-7

**Published:** 2023-07-22

**Authors:** Mogge Hajiesmaeil, Francesco Ravasini, Flavia Risi, Giorgia Magnarini, Anna Olivieri, Eugenia D’Atanasio, Hamid Galehdari, Beniamino Trombetta, Fulvio Cruciani

**Affiliations:** 1 Department of Biology and Biotechnologies ‘Charles Darwin’, Sapienza University of Rome, P.le Aldo Moro 5, 00185 Rome, Italy; 2 Department of Biology and Biotechnology ‘Lazzaro Spallanzani’, Pavia University, Pavia, Italy; 3 NBFC, National Biodiversity Future Center, 90133 Palermo, Italy; 4 Institute of Molecular Biology and Pathology (IBPM), CNR, 00185 Rome, Italy; 5 Department of Biology, Faculty of Sciences, Shahid Chamran University of Ahvaz, Ahvaz, Iran

**Keywords:** Computational biology and bioinformatics, Evolution, Genetics

## Abstract

The ampliconic region of the human Y chromosome consists of large duplicated sequences that can undergo non-allelic homologous recombination (NAHR), resulting in structural rearrangements that may cause infertility, especially when they occur in the azoospermia factor b/c (AZFb/c) region. Although AZF duplications have long been neglected due to the technical limitations of STS-based studies that focused mainly on deletions, recent next generation sequencing (NGS) technologies provided evidence for their importance in fertility. In this study, a NGS read depth approach was used to detect AZFb/c rearrangements in 87 Iranians from different ethnic groups. The duplication frequency in Iran proved to be twice as high as in the "1000 Genomes" dataset. Interestingly, most duplications were found in patrilineal ethnic groups, possibly as a consequence of their lower male effective population size which can counteract negative selection. Moreover, we found a large 8.0 Mb duplication, resulting in a fourfold increase in the copy number of AZFc genes, which to our knowledge is the largest duplication ever reported in this region. Overall, our results suggest that it is important to consider not only AZF deletions but also duplications to investigate the causes of male infertility, especially in patrilineal clan-based populations.

## Introduction

**Figure 1:**
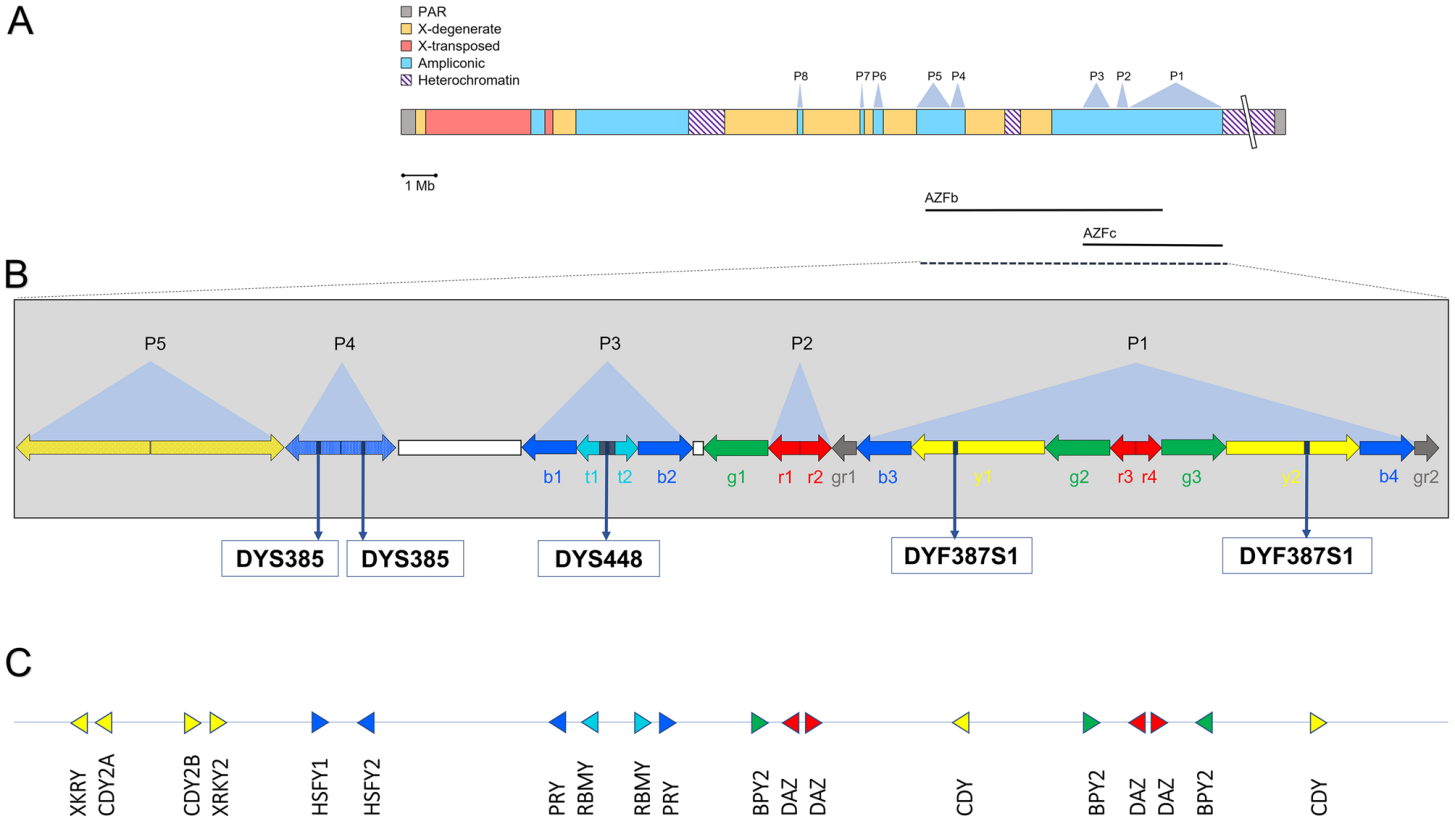
The Y chromosome AZFc region. (**A**) Y chromosome structure, showing the location of the AZFb and AZFc regions. (**B**) Magnification of the AZFc region. Multiple copies of six amplicons are represented by colour-coded arrows (b—blue, t—teal, g—green, r—red, y—yellow, and Gr—grey; adapted from Teitz et al.^9^, not in scale). The arrow's direction indicates amplicon copy orientation. Most of the amplicons are arranged to form P1, P2, and P3 palindromes. The dark grey rectangle between t1 and t2 represents the large P3 spacer, while the white rectangle between b2 and g1 represents a single copy of Inverted Repeats 1 (IR1). Approximate positions of DYS448, DYF387S1 and DYS385 short tandem repeats are shown. (**C**) Locations of protein coding genes within the AZFc. Triangle orientation refers to 5′–3′ polarity.

The ampliconic portion of the male specific region of the human Y chromosome (MSY) consists of large duplicated sequences, named amplicons most of which are arranged in eight large palindromes (P1–P8; Fig. 1)^1,2^. Owing to the high sequence identity shared between the amplicons, non-allelic homologous recombination (NAHR) frequently occurs between these structures. Although in most cases these events are resolved by gene conversion^3–7^, they can sometimes induce structural rearrangements such as inversions, duplications and deletions and, therefore, copy number variants (CNVs) of the amplicons^8–10^.

The phenotypic effects of these rearrangements are variable, and they may also be influenced by other genomic regions and environmental factors^11^. The vast majority of the MSY amplicon rearrangements that lead to known phenotypic effects involve two large and partially overlapping regions called Azoospermia Factor b and c (AZFb and AZFc), (Fig. 1)^12^, which contain most of the MSY genes with a testis-specific expression pattern^13^. AZFc harbours five protein coding gene families (PRY [MIM: 400019, 400041], RBMY [MIM: 400006], BPY2 [MIM: 400013], DAZ [MIM: 400003], and CDY [MIM: 400016]), which are likely to be dosage sensitive^9,14^. Usually, complete deletion of the AZFb or AZFc is associated with spermatogenic problems (azoospermia or oligozoospermia), and is considered one of the most common genetic factors causing male infertility^15–20^. Less extensive deletions can similarly lead to reduced male fertility or various pathologies^21–23^. However, it is important to note that in most cases, these rearrangements do not appear to have any effect on the phenotype of the individual carrying them, and their prevalence in the global population is therefore not completely understood^9^. In addition, by comparing different populations, the prevalence and the effect of these rearrangements may greatly vary, especially considering different Y chromosome haplogroups and ethnic backgrounds.

The analysis of structural rearrangements in the MSY ampliconic region has always been an important concern, both from a screening and clinical perspective, due to its importance in male infertility (especially for the AZFb and AZFc regions)^18^. Recently, there has also been a rising interest in the molecular characterization of these rearrangements using sequencing technologies due to its potential in clarifying their molecular mechanisms and evolutionary implications^9,10^. One of these studies comprehensively investigated these topics by analysing the 1,216 male samples from the 1000 Genomes Project^9^. However, the molecular characterization of the Y chromosome amplicon rearrangements for many different human populations around the world has not yet been studied, especially for NAHR events that do not lead to pathological conditions.

Y chromosome amplicon rearrangements may also be relevant for forensic genetics analysis. Some of the short tandem repeats of the Y chromosome (Y-STRs) commonly employed for forensic purposes are located within amplicons or in a region between them and are therefore potentially prone to duplications or deletions^10,24–30^. Molecular characterization of structural rearrangements affecting these loci may help to better interpret Y-STRs profiles (see for example the locations of the Y-STRs DYS385, DYS448 and DYF387S1 in the AZF region, Fig. 1).

A recent cross-sectional study reported that the highest rate of primary male infertility in the world is in Middle Eastern countries, with an upward trend over the last 3 decades^31^. Iran is the second largest country in the Middle East and spans more than ten different ethnic groups, characterised by different rates of endogamy and patrilineality. Thus, a genetic study that takes into account sub-population structuring is needed to accurately assess the prevalence of male genetic abnormalities linked to infertility. Notably, to date, the molecular characterization of Y chromosome rearrangements in Iran has been poorly explored and most of the studies are mainly focused on deletions rather than duplications^32–35^.

In the present study, we report the first unbiased molecular characterization of the AZFb/c structural rearrangements in Iran. We performed a sequence read depth analysis (mean whole genome depth > 30 ×) of the ampliconic region of 87 individuals belonging to 8 well-defined ethnic groups, without considering any pathological records. We found an incidence of duplications in the AZFb/c region significantly higher than the 1000 Genome Project worldwide population. Although most of the patterns we identified have already been observed, we found two interesting cases of two subsequent NAHR events for the first time to our knowledge, one of them leading to the retention of a very high number of amplicons in the AZFc region. Moreover, we have identified a chromosomal rearrangement that may be specific to a particular nomadic ethnic group characterised by patrilocality and endogamy. This project provides valuable information about Y chromosome rearrangements among different ethnic groups in Iran, which can be extremely helpful for health care providers in planning appropriate population-based treatment and prevention strategies.

## Results

### Identification of CNVs in the ampliconic region

**Figure 2:**
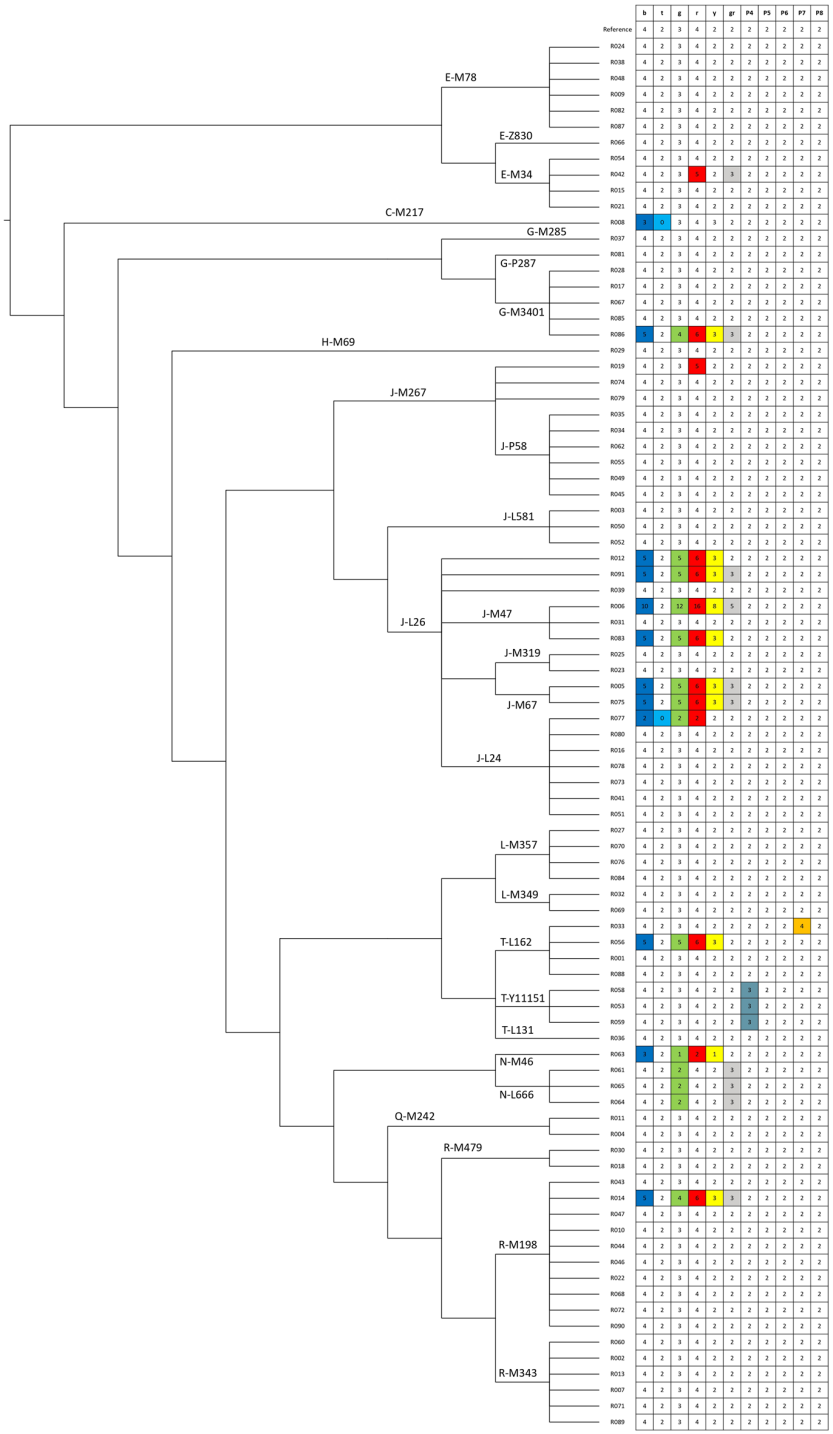
Phylogenetic tree of the 87 males analysed. For each subject the number of copies of each amplicon is shown and compared to the reference. CNVs are colour coded.

**Figure 3:**
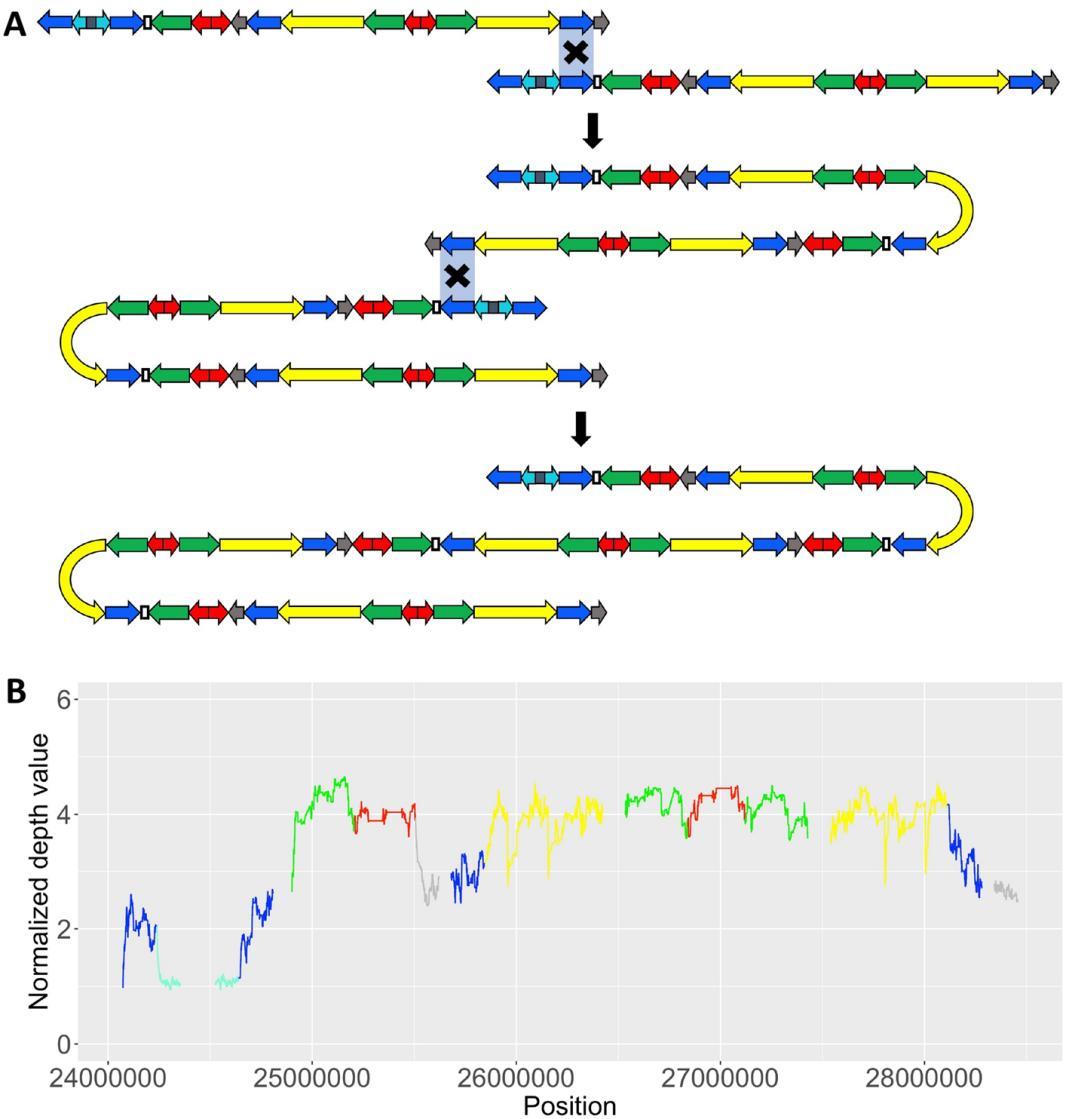
(**A**) Proposed NAHR events to explain the pattern observed in the sample R006: two subsequent b2/b4 duplications. (**B**) Normalised EMA value of the depth in the AZFc of R006. The colours correspond to the amplicon names (blue, teal, green, red, grey and yellow). Empty spaces and precise horizontal lines indicate regions that were not included in the analysis like repetitive elements, DAZ genes or regions in-between two amplicons.

To detect CNVs in the amplicon region of the Y chromosome in the analysed samples (Supplementary Table 1), we normalised the mean depth values of the selected amplicons by the mean depth value of a unique region of the Y chromosome (Supplementary Tables 2 and 3)^9,10^. The resulting copy number calls are based on the expected value of the ratio between the copy number of the putative amplicon and the copy number of the reference genome (Fig. 2 and Supplementary Tables 4 and 5). Note that the reference sequence carries the ancestral number of amplicons^9^ and therefore chromosomes showing differences in copy number compared to the reference have been interested by mutational events. We focused on the entire AZFc region (which includes palindromes P1, P2 and P3, as well as other segmental duplications), palindromes P4 and P5 of the AZFb region, and palindromes P6–P8 (Fig. 1). In this way, we identified a total of 21 males (24.1%) with at least one amplicon with a copy number different from the reference. In particular, we observed 15 chromosomes with duplications, 2 chromosomes with deletions and 4 chromosomes carrying both deletions and duplications. Most CNVs (17 out of 21) were found within the AZFc region. As for the amplicons in this particularly complex region, we confirmed our observations with the normalised EMA analysis (Figs. 2, 3B and Supplementary Fig. 1). The proportion of Iranian individuals with structural rearrangements is higher than in the global population represented by the 1000 Genomes (24.1% vs. 16.0%), although this difference is not statistically significant (Fisher exact test p = 0.053). Interestingly, when deletions and duplications were considered separately, we observed a much higher and statistically significant incidence of duplications in the Iranian population (17.2% vs. 7.8%, p = 0.005) than in the global population and, conversely, a lower incidence of deletions (2.3% vs. 6.9%, p = 0.116).

### Description of recombinational events

Some of the structural rearrangements identified here are easily explained by a single NAHR event occurring between amplicons with the same orientation. However, there are several cases for which more complex rearrangements are required to explain the observed pattern.

Surprisingly, we identified a large duplication (about 8.0 Mb) in sample R006 (haplogroup J-M47), resulting in a fourfold increase of the copy number of the genes BPY2, DAZ and CDY1 and possibly two additional copies of PRY (Figs. 2, 3). To our knowledge, this is the largest duplication ever reported in the AZFc region This rearrangement is compatible with two NAHR events that have occurred between sister chromatids. Indeed, two subsequent b2/b4 duplications are required to explain this pattern (Fig. 3). Notably, genes in this region have been proven to be dosage sensitive, therefore their duplication could reduce fertility, at least in Asian men^14^.

Amplicon copy number in samples R005, R075 and R091 indicates a complex evolutionary history (Fig. 2). Their rearrangement pattern is compatible with a series of two subsequent NAHR events between amplicons, for example a common r2/r3 inversion^36^ followed by a b2/b3 duplication, but these cannot explain the presence of an extra grey amplicon. We argue that an additional duplication event occurred involving this amplicon (Supplementary Fig. 2). This complex pattern has been previously observed in only one individual of haplogroup O2^9^. In our data set, this pattern is present in one J-L26* individual and two males belonging to haplogroup J-M67. The phylogenetic relationships of the two J-M67 males with respect to eighth J-M67 subjects analysed by Teitz et al.^9^, which do not carry the rearrangement, suggest that this mutation could be polyphyletic within J-M67 too. On the other hand, three individuals, namely R012, R056 and R083 show the same simple NAHR pattern, without the extra grey amplicon (Fig. 2, Supplementary Fig. 2). This arrangement of amplicons is relatively common and has been previously observed throughout the whole Y chromosome phylogenetic tree in the haplogroups A0, C1, C3, O2, O3 and R1b among the 1000 Genomes samples^9^. Consistently with its polyphyletic nature, we found it in three additional haplogroups (J-L26*, J-M47 and T-L162; Fig. 2).

The pattern found in the sample R008 (haplogroup C-M217) can be explained by two simple NAHR events: a g1/g2 duplication and a b1/b3 deletion (Fig. 2, Supplementary Fig. 3). In this case, the two events may have occurred at the same moment, since the deletion can be both an inter-chromatic and an intra-chromatic event. This amplicon pattern has not been observed in the 1000 Genomes dataset as reported by Teitz et al.^9^.

All the samples that we detected as haplogroup N belong to the Turkmen ethnic group and have some kind of chromosome rearrangements in the AZFc region (Fig. 2). The sample of haplogroup N-M46 (R063) has an amplicon copy number that is compatible with a r2/r3 inversion followed by a b2/b3 deletion (Supplementary Fig. 4A). This rearrangement has been previously reported to be fixed in haplogroup N3 chromosomes^37^. The three samples of haplogroup N-L666 (R061, R064 and R065) show a pattern that can be explained by the two events described above and a subsequent duplication event occurred between the blue amplicons (Supplementary Fig. 4B). This event, described as b2/b3 rescue, has already been observed among haplogroup N individuals (Supplementary Fig. 5) and can be interpreted as a way to partially or completely restore the dosage of genes PRY, DYZ, CDY and BPY2 present in the amplicons^9^.

The pattern observed in the sample R077 (haplogroup J-L24) is partially compatible with a b1/b3 deletion, but the presence of an additional grey amplicon (2 instead of 1) suggests that a complex non-NAHR event occurred to explain the arrangements of the amplicons.

The individuals R014 and R086, belonging to two different haplogroups (R-M198 and G-M3406, respectively) show an arrangement that can be explained by a simple NAHR recombination between two green amplicons resulting in a large duplication known as gr/gr duplication, (+ 1 b, + 1 g, + 2 r, 1 gr, + 1 y). This pattern has previously been reported as the most common AZFc rearrangement and has been observed in several Y chromosome haplogroups as a consequence of multiple independent recombinational events^9^. Based on previous studies, men carrying the gr/gr primary duplication are at increased risk for infertility in Asia^14^ while this association seems to be not statistically significant among European populations^38,39^.

The individuals R019 (haplogroup J-M267), R033 (Haplogroup T-L162), R042 (haplogroup E-M34), show three different patterns that are not compatible with simple NAHR events occurring between amplicons. They can only be explained by micro-duplications that involve only one amplicon (for samples R019 and R033) or two amplicons (for the sample R042) (Fig. 2, Supplementary Table 6). All these patterns have already been previously observed, although in different Y chromosome backgrounds^9^.

Notably, individuals R053, R058 and R059 share the same extra copy of P4 palindrome arm in the AZFb region, which may result in an additional HSFY gene. These samples belong to the same haplogroup (T-Y11151) and ethnic group (Qashqai), suggesting that this mutation occurred only once in this population and likely spread due to patrilineality. Although this pattern has also been observed in one individual of haplogroup T from the 1000 Genome Project^9^, it belongs to a paraphyletic haplogroup suggesting that two different independent recombinational events occurred. Since the well-known multicopy Y-STR DYS385 is located within amplicon P4, we performed a Y-STR analysis among 93 Qahsqai individuals belonging to 9 different clans as a fast experimental approach to evaluate the frequency of this duplication within this ethnic group. The three sequenced subjects carrying the P4 duplication showed an unbalanced Y-STR pattern clearly due to the presence of an extra copy of the DYS385 Y-STR (Supplementary Fig. 6). The same pattern was also observed in 19 additional subjects, all belonging to a specific Qashqai clan, representing 56.4% (22 out of 39) of the males (Supplementary Table 7).

## Discussion

Structural rearrangements in the ampliconic region of the human Y chromosome are frequent and can be found in every major haplogroup of the Y phylogeny^9^. Most of these recombinational events do not lead to evident phenotypic traits and therefore the number and relevance of these variations may have been underestimated in hospital-based studies, leading to biased reporting^16,21,22,40^. Furthermore, most of previous studies have been performed using the sequence-tagged sites approach that can only detect deletions and not duplications^41^. By using high-throughput sequencing technologies, now it is possible to find the real extent of both deletions and duplications and identify particular structures of the Y chromosome^9,10,36^. Although AZF duplications have been long neglected in human molecular reproduction reports, mainly because of technical limitations of the STS-based approach, there is an increasing number of studies reporting a significant association between fertility and different AZF duplications^19,42^. On a theoretical basis, duplications of the AZF regions can affect fertility either leading to an excess of gene products or causing an imbalance among factors involved in spermatogenesis. Indeed, the AZFc region carries different coding and non-coding gene families^1^, at least one of which (DAZ family) seems to be dosage sensitive^18,43^. Accordingly, the gr/gr duplication, leading to an increase of DAZ genes, has been repeatedly linked to infertility in Asian men^14^, although such an association has not been replicated in Europeans^38^.

In this study, for the first time, we analysed the copy number variants in the Y chromosome ampliconic region through an NGS-based read depth approach on 87 Iranian individuals belonging to different ethnic groups, who have been randomly selected without considering any pathological records. We identified 21 samples with at least one amplicon that had a copy number different from the reference sequence, for a total of 12 different rearrangements attributable to 17 mutational events (Fig. 2, Supplementary Table 6). Surprisingly, the frequency of Y chromosome duplications in the Iranian population is twice higher than in the 1000 Genomes dataset^9^, which can be considered as a proxy to the world population (17.2% vs. 7.8%, p = 0.005). However, to confirm this higher frequency of duplications in the Iranian populations further studies with larger sample sizes are needed. This finding raises the possibility that duplications in the Iranian populations can play a role in the high incidence of primary infertility reported in Iran^44^ and, in general, in the Middle Eastern populations^45^, although this hypothesis cannot be formally tested in this study, which lacks clinical information.

It is interesting to note that in our study most duplications were found in ethnic groups organised in patrilineal clans (Bakhtiari, Turkmen and Qashqai). Since patrilineality implies a lower male effective population size^46,47^, leading to a higher impact of genetic drift which can counteract selection, this type of social structure could play an important role in the spread and maintenance of Y chromosome rearrangements, despite their possible impact on fertility. Consistently, we have observed a high frequency of duplications among the Turkmen and the Qashqai, both Turkic-speaking peoples, for whom a strong patrilineal social organisation has been reported^48^. Among the Turkmen, we report a relatively common b2/b3 rescue rearrangement associated with haplogroup N (3 out of 9 Turkmen, 33.3%), which was rarely found in other populations worldwide^9^. Among the Qashqai, we identified three individuals belonging to the same haplogroup (T-Y11151) and carrying one extra copy of the P4 palindrome arms (frequency 3 out of 16, 18.7%). When we extended our analysis by adding additional Qashqai males (93 individuals in total), genotyped at the DYS385 Y-STR located in the P4 palindrome, we observed the P4 duplication in other 19 Qashqai, all belonging to the same clan (clan-specific duplication frequency 56.4%, Supplementary Table 7). We hypothesise that a duplication event occurred in a branch of T-Y1115 and spread in a particular clan thanks to the patrilineal social organisation of the Qashqai. Amplicon copy number variations in the human Y chromosome are limited by natural selection^9^. Since many genes related to spermatogenesis are present in amplicons, deletions that completely remove one or more of these genes have deleterious effects on fitness. Therefore, it is common to observe, along the Y chromosome phylogeny, duplication events occurring subsequently to deletion ones, to restore or (more commonly) to partially restore the correct amplicon number. An example of this event can be observed in the Turkmen individuals of haplogroup N presented here. However, recently it has been hypothesised that the reverse is also possible: a deletion may occur after a duplication to avoid an incorrect gene dosage^10^. We identified a possible example of this sequence of events in a male Bakhtiari (sample R008), as the observed pattern is compatible with a g1/g2 duplication followed by a b1/b3 deletion. In this case, after the deletion, some amplicons retained the same amount of copy they had after the duplication (e.g., the yellow amplicon) and one was completely lost (teal), while the red and green amplicons returned to their original state.

Notably, in another Bakhtiari individual (R006) we identified a large duplication (approximately 8.0 Mb) with an impressive number of copies for most amplicons in the AZFc, as the result of two subsequent b2/b4 duplications, resulting in a fourfold increase in copy number for the BPY2, DAZ and CDY1 genes (from 3, 4 and 2 copies to 12, 16 and 8 copies, respectively). Some of these genes have been reported to be dosage sensitive and their increased expression may interfere with normal spermatogenesis, leading to infertility or reduced fertility. This structure should lead to incorrect gene dosage and therefore be unfavoured by natural selection^10^. It is important to note that this is the largest duplication ever reported in the AZFc region. However, we cannot make any firm assumptions about the role of this rearrangement in fertility because we have no clinical information on this individual.

Further studies regarding the structural rearrangements of the human Y chromosome are needed to shed light on the evolutionary processes that shaped them. Overall, the results presented here suggest that it is important to consider not only AZF deletions but also duplications when investigating for the causes of male infertility, especially in patrilineal, clan-based populations.

## Material and methods

### The sample

The male individuals analysed in this study were from different regions of Iran, from different cities and rural areas (Supplementary Table 1). We selected only unrelated individuals on a voluntary basis, without considering any pathological records, and if they were the results of consanguineous marriages or not. After providing informed consent, saliva was collected from the participants using the non-invasive Oragene collection kit (DNA Genotek). DNA was extracted according to the manufacturer's instructions. The present project is part of a broader study concerning the genomic diversity of Iranian populations that obtained approval from the “National Committee for Ethics in Biomedical Research" of Iran, issued by the Shahid Chamran University of Ahvaz-Iran (Certification number: IR.NIMAD.REC.1395.003). All methods were performed in accordance with the relevant guidelines and regulations.

### Whole genome sequencing

The DNA samples were amplified by ligation-mediated PCR, after random fragmentation. DNBseq platform was used to sequence the paired-end reads and the base-calling was performed with the default parameters of the DNBseq base calling Software. The alignment against the human reference genome (GRCh37/hg19) was carried out with Burrows-Wheeler Aligner (BWA) software^49^. All the processing steps including library preparation, sequencing and alignment were performed by Beijing Genomics Institute (BGI, Hong-Kong). The average resulting sequencing depth for each sample is reported in supplementary table 1.

### Y haplogroup assignment

To assess the phylogenetic relationships among different chromosomes carrying CNVs, we determined the Y chromosome haplogroup affiliation for each sample using the tool pathPhynder^50^, with standard parameters, using the complete sequence of the Y chromosome of these individuals. We used the reference tree provided in Martiniano et al.^50^, (https://github.com/ruidlpm/pathPhynder), which encompasses the vast majority of the human Y chromosome variability with a total of 2014 individuals included. For the purpose of this study, we have simplified the phylogenetic relationships obtained, as shown in Fig. 2 and Supplementary Table 1, by highlighting only the major Y chromosome haplogroups and defining markers.

### Detection of copy number variations in the MSY

To identify copy number variations (CNVs) in the MSY, we employed the methods described in Teitz et al.^9^ and Ravasini et al.^10^. Briefly, we obtained the depth value for the entire AZFc region (which includes palindromes P1, P2 and P3 and other repetitive DNA segments), palindromes P4 and P5 within AZFb and palindromes P6-P8. Using samtools^51^, we also obtained the depth value for a 900 kb single copy region for the Y chromosome, which was used for normalisation. Since the copy number of interspersed repetitive elements varies considerably in the human genome, their presence in a depth analysis may alter the actual number of amplicons found. Therefore, these elements as well as simple sequence repeats were excluded from the depth calling in the palindromes with the Table Browser tool of the UCSC Genome Browser (https://genome.ucsc.edu/). We also excluded the DAZ genes present in the red amplicons, as they could be interested by partial CNVs and then alter the number of copies observed in the red amplicon (for the final regions analysed see Supplementary Table 2). Then, for each sample, we normalised the mean depth of each amplicon by the mean depth of the single copy region. Using this method, an amplicon with the same copy number as the reference sequence has a normalised depth value of ≈ 1. On the other hand, if there is a variation in the number of copies, the normalised depth value is ≠ 1 and it is proportional to the effective number of copies of that amplicon. The normalised depth value thresholds to call different CNVs were set by the midpoints between the expected depth values for each amplicon^9^ (Supplementary Table 4). The results obtained using this method have been proven to be consistent, since they have previously been validated with FISH analysis of cells^9^.

As in Ravasini et al.^10^, we also performed a normalised Exponential Moving Average (EMA) analysis to confirm the results obtained with the previous method, for the AZFc region. We calculated the EMA of the depth value in the amplicons using sliding windows of 10 kb moving by 1 bp. The EMA values obtained for each position were then normalised by the mean depth value of the 900 kb single region of the same sample. In this way, the value obtained should indicate the ratio between the copy number of the sample and the reference as in the method mentioned above. However, we note that the values may be fluctuating due to random differences in the sequencing and to the presence of different copy numbers compared to the reference sequence in the adjacent amplicons.

Fisher's exact test was used to compare the incidence of CNVs found in our sample with those of the 1000 Genomes samples analysed by Teitz et al.^9^. This comparison did not take into account the CNVs found by Teitz et al.^9^ in the IRs sequences, which were not analysed in the present studies.

R scripts and other information used to perform these analyses are available in the GitHub repository AZFinder at this link https://github.com/fravasini/AZFinder.

### Y-STR genotyping

Multiplex amplification of Y-STRs was performed on an Applied Biosystem® Veriti™ 96‐well thermal cycler (ThermoFisher Scientific) by using the Yfiler™ Plus PCR Amplification Kit (ThermoFisher Scientific) according to the manufacturer's protocol utilising 1 ng of genomic DNA. The amplified DNAs were then electrophoresed on the 24-capillary Applied Biosystems® 3500xL Genetic Analyzer (ThermoFisher Scientific) and the fragment analysis was performed using GeneMapper® ID-X software v.1.4 (ThermoFisher Scientific).

## Supplementary Information

Supplementary Figures.

Supplementary Tables.

## Data Availability

The data analysed in this study are deposited in the European Nucleotide Archive (ENA, https://www.ebi.ac.uk/ena/browser/home) under Accession Number PRJEB61684. Detailed information and R scripts used to perform the analyses are available in the GitHub repository AZFinder at this link https://github.com/fravasini/AZFinder.
