# Supplementary material for: High incidence of AZF duplications in clan-structured Iranian populations detected through Y chromosome sequencing read depth analysis
Source: Sci Rep. 2023 Jul 22;13:11857. doi: 10.1038/s41598-023-39069-7 (PMC10363161; doi:10.1038/s41598-023-39069-7)
Supplement: Supplementary file 1 — Supplementary Figures. [file 41598_2023_39069_MOESM1_ESM.pdf]

Supplementary figure 1.

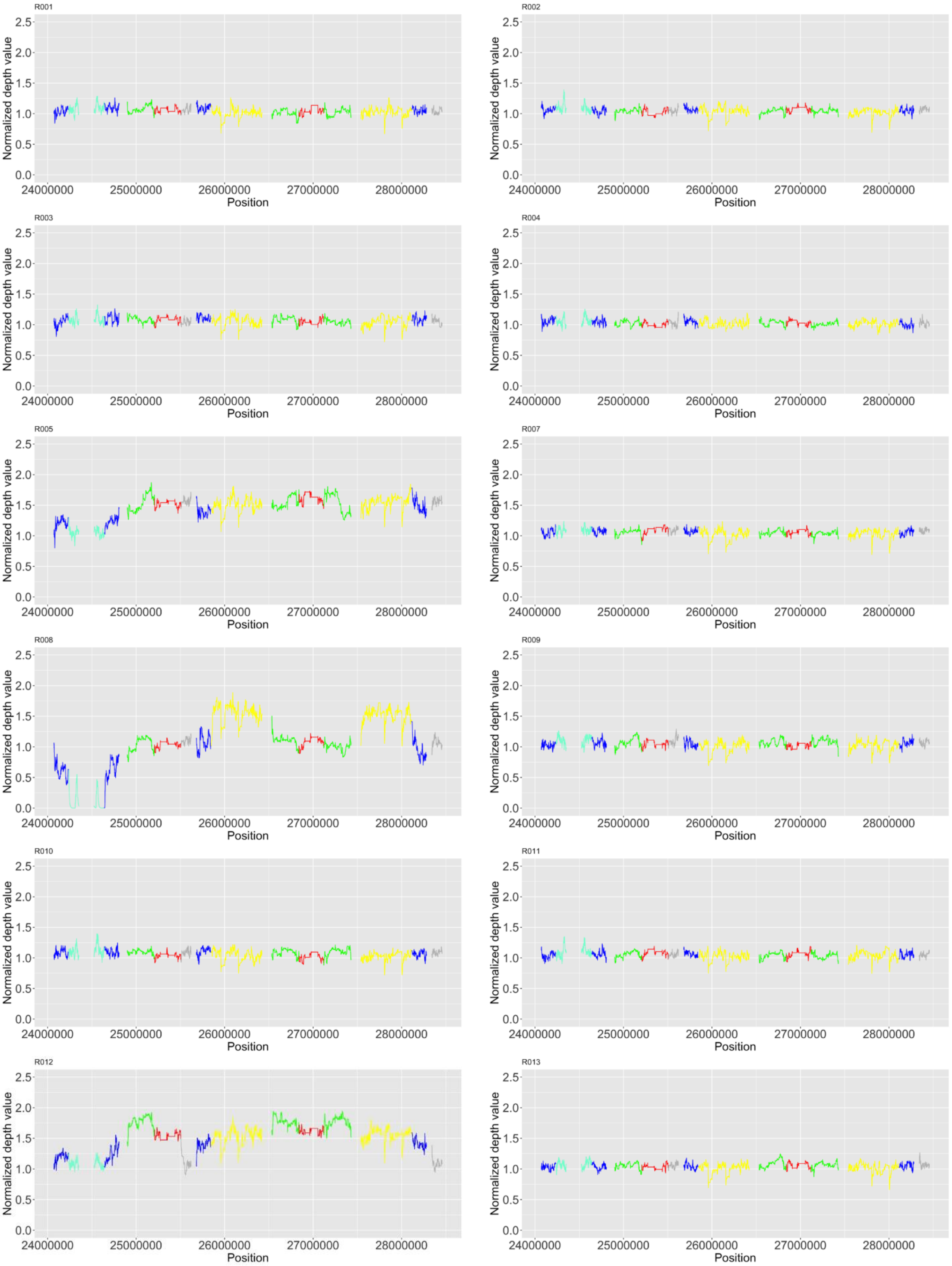

Supplementary figure 1.

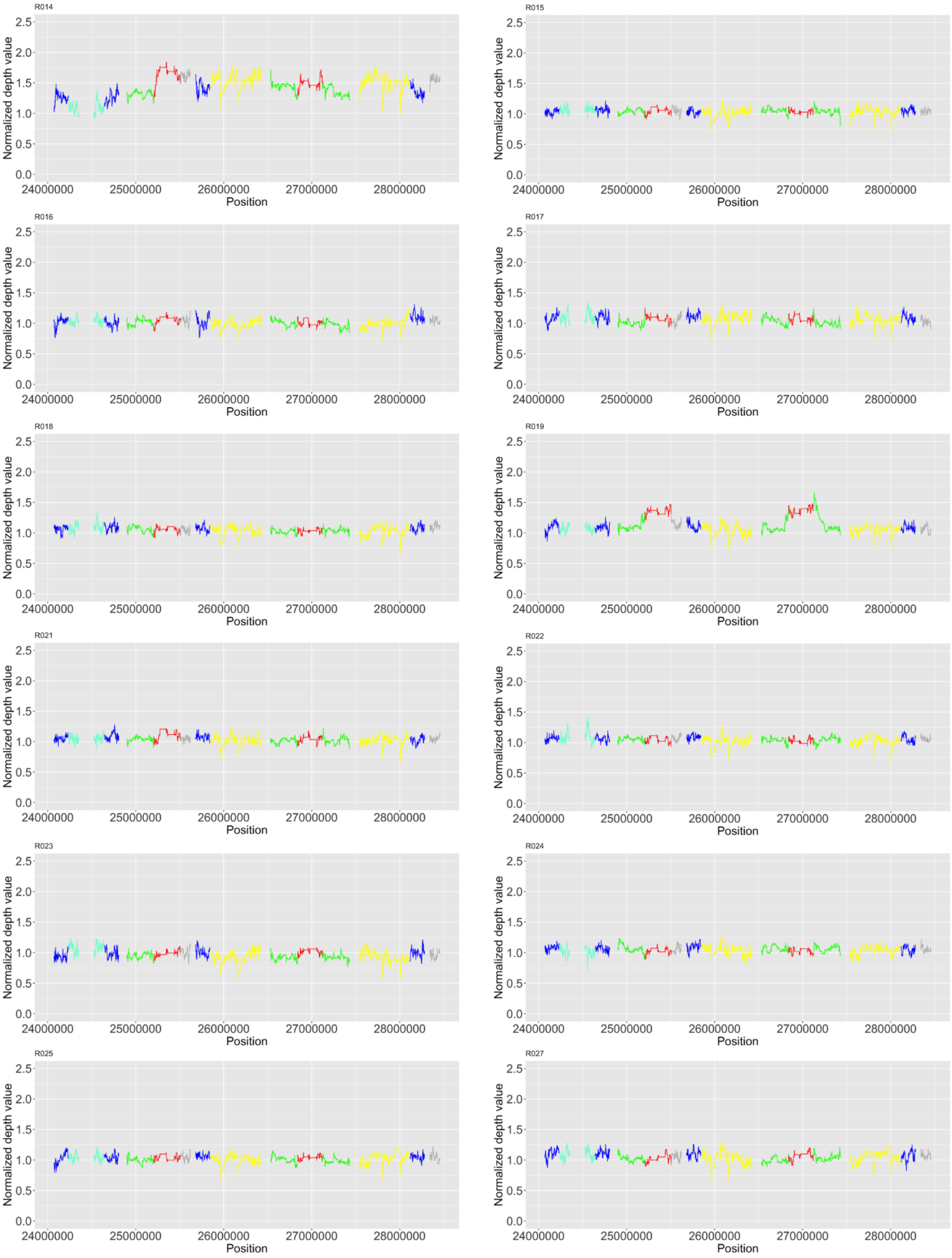

Supplementary figure 1.

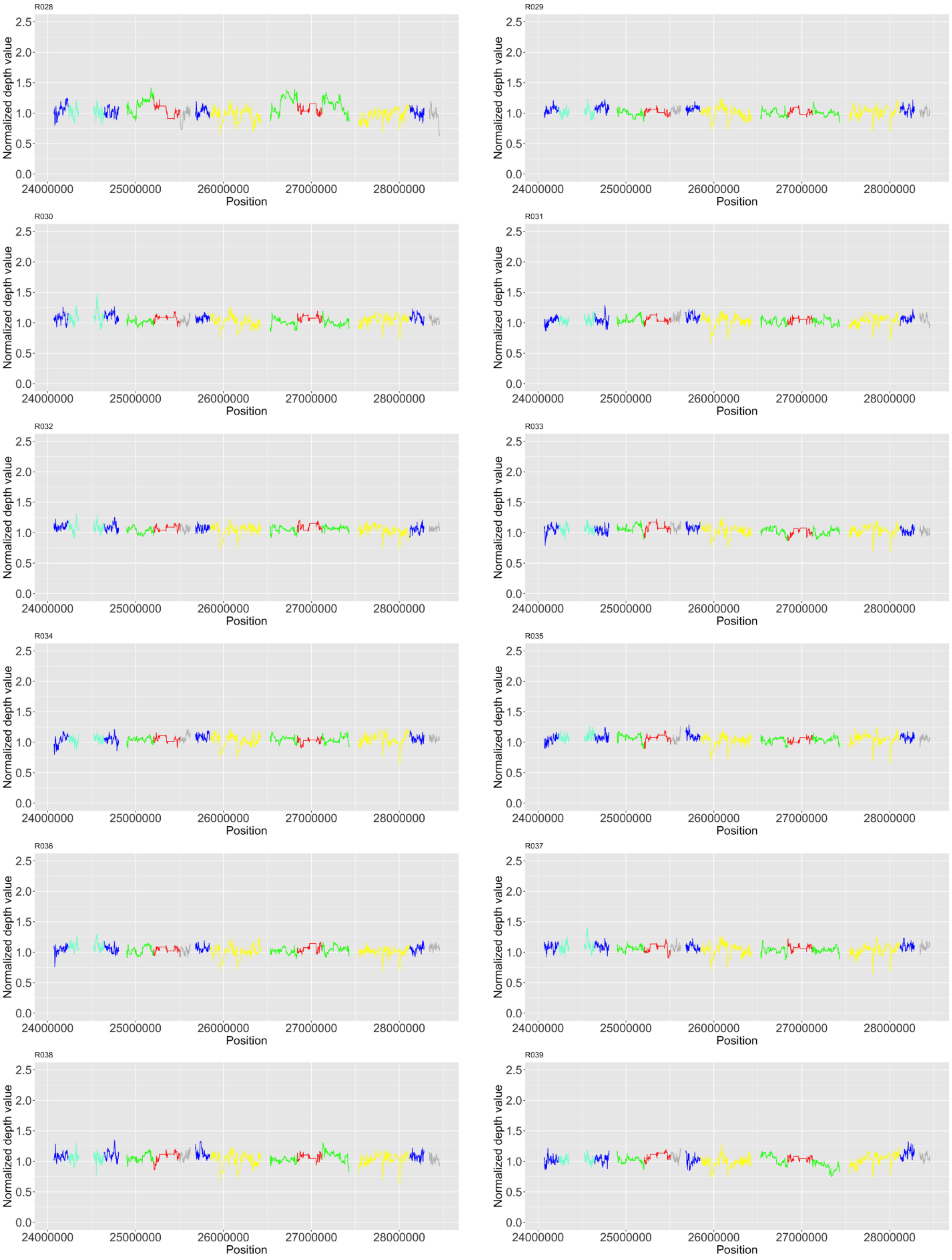

Supplementary figure 1.

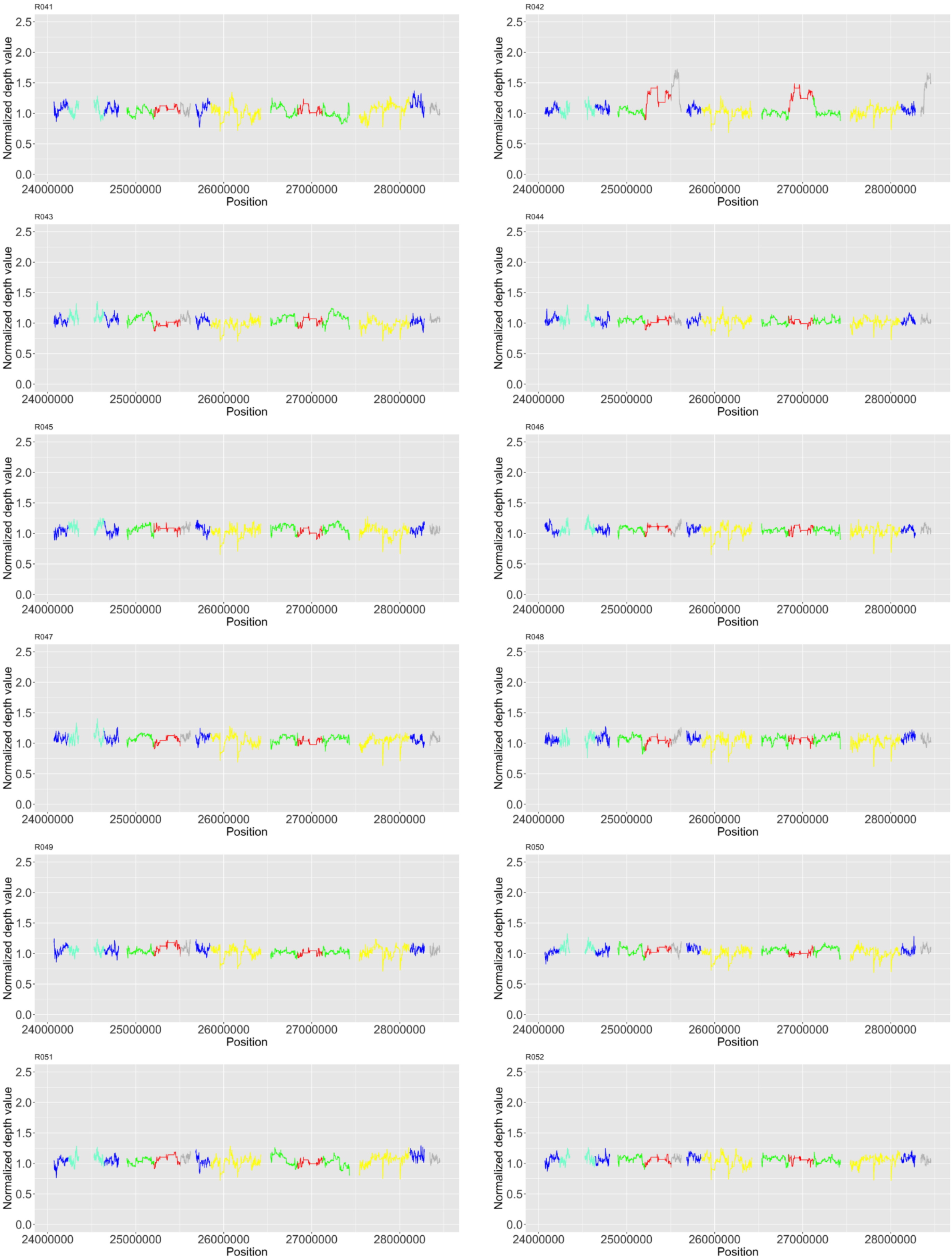

Supplementary figure 1.

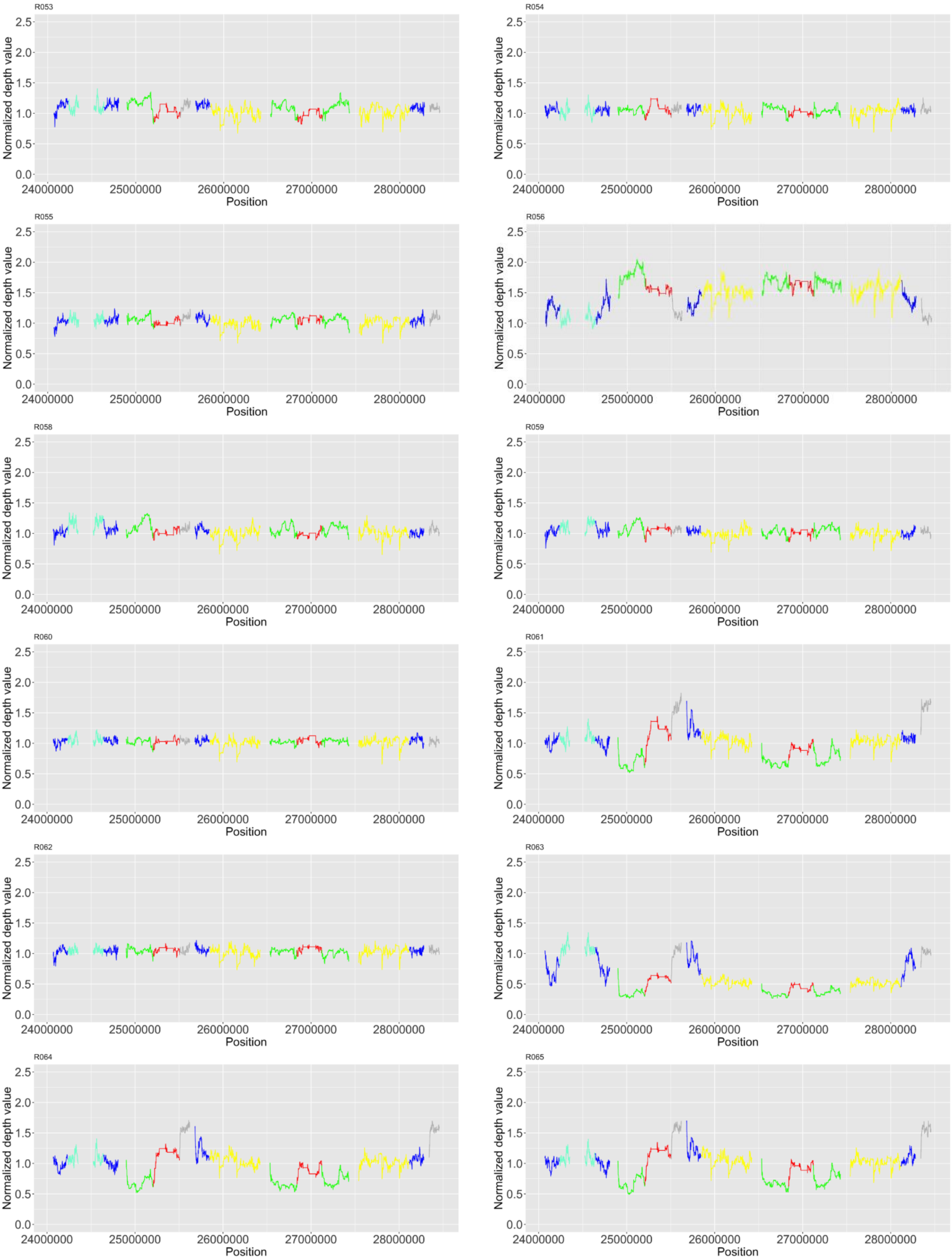

Supplementary figure 1.

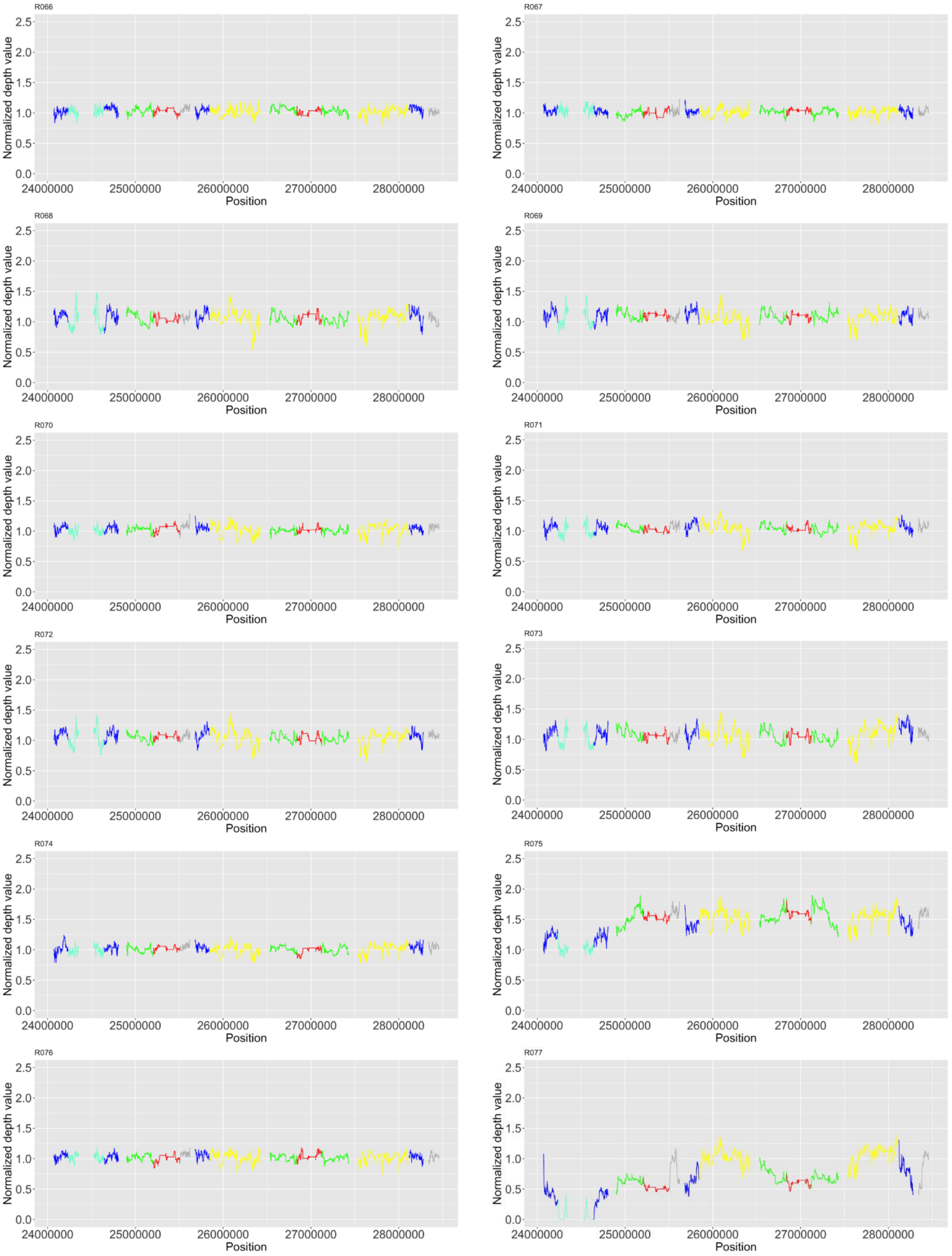

Supplementary figure 1.

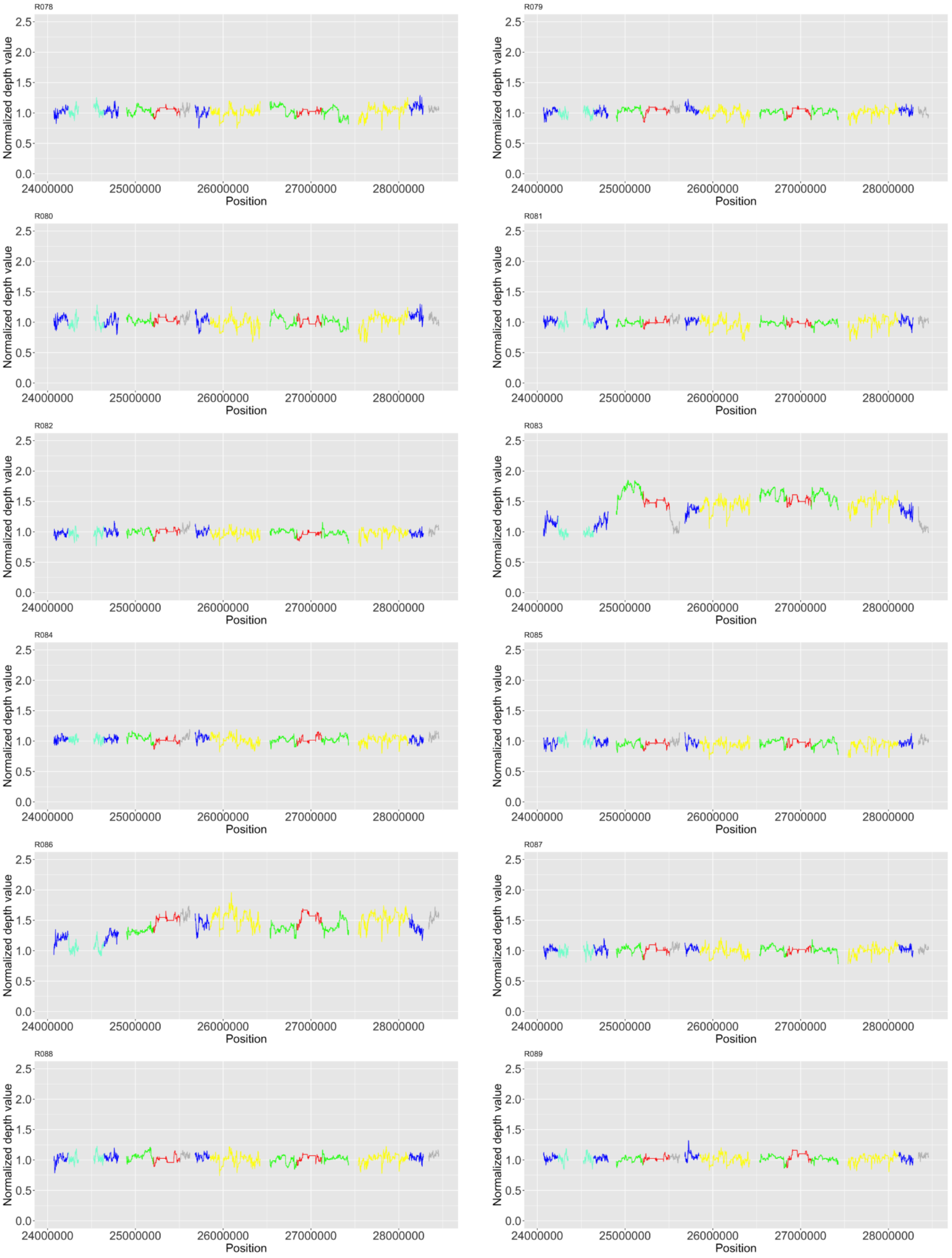

Supplementary figure 1.

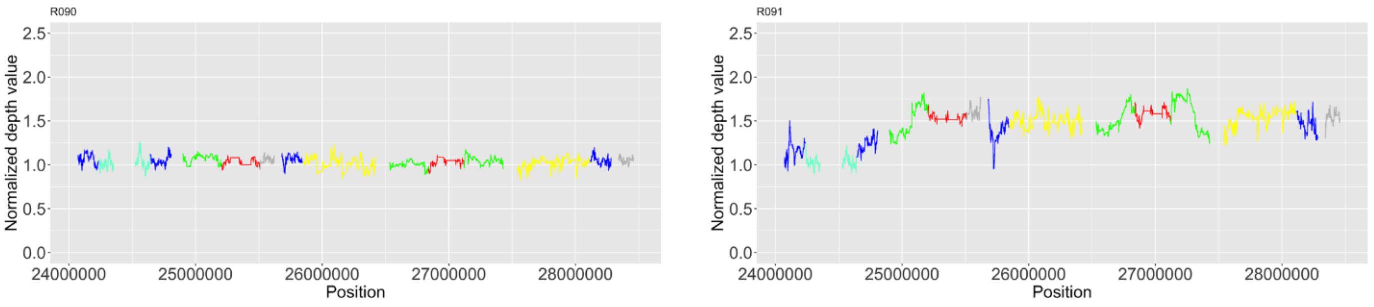

Supplementary figure 1. Normalized EMA value of the depth in the AZFc region for each sample (excluding R006). The colors correspond to the amplicon names (blue, teal, green, red, gray and yellow). Empty spaces and precise horizontal lines indicate regions that were not included in the analysis because like repetitive elements, DAZ genes or regions in-between two amplicons.

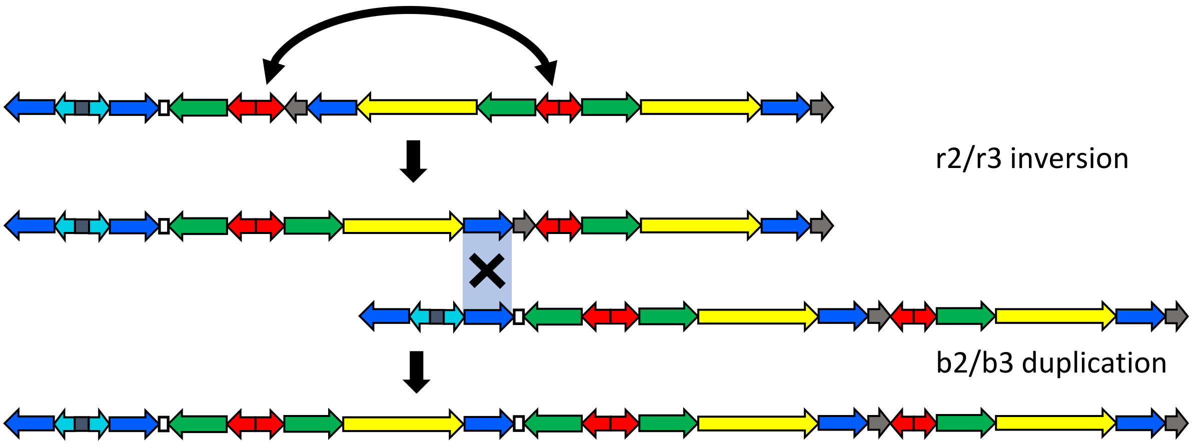

Supplementary figure 2. Possible recombinational events that lead to the amplicon pattern in the samples R005, R012, R056, R075, R083 and R091: a b2/b3 duplication after a r2/r3 inversion. For R005, R075 and R091 an additional complex event leading to an extra copy of the gray amplicon is necessary to explain the pattern observed.

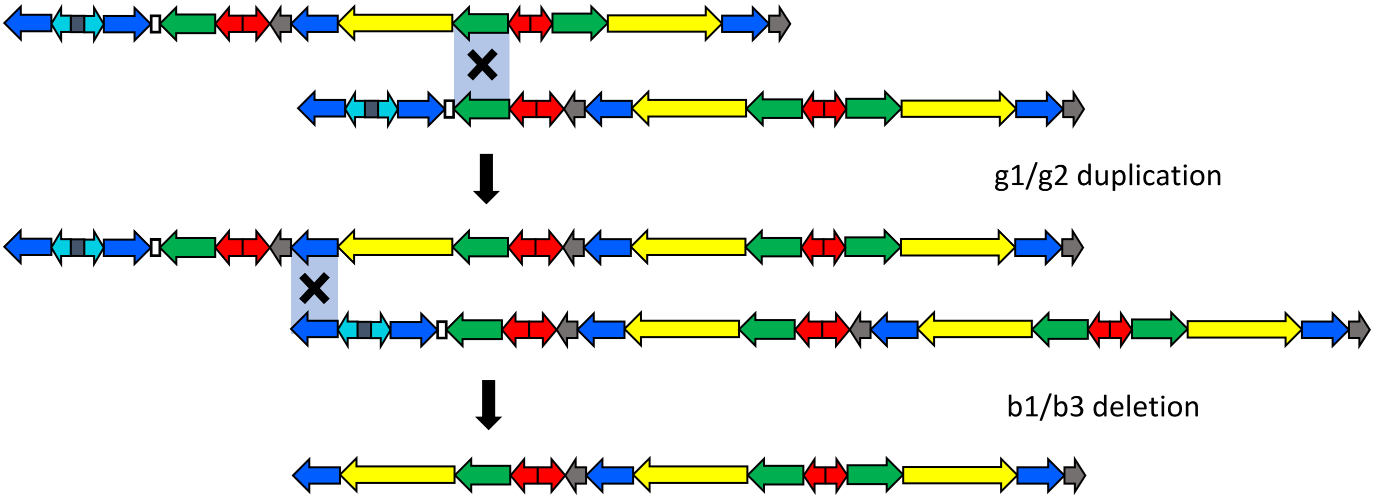

Supplementary figure 3. NAHR events between AZFc amplicons that led to the pattern observed in the sample R008: a g1/g2 duplication followed by a b1/b3 deletion. Note that the b1/b3 deletion, although represented as an inter-chromatid event, could also have occurred within the same chromatid. Moreover, the two events could have occurred simultaneously.

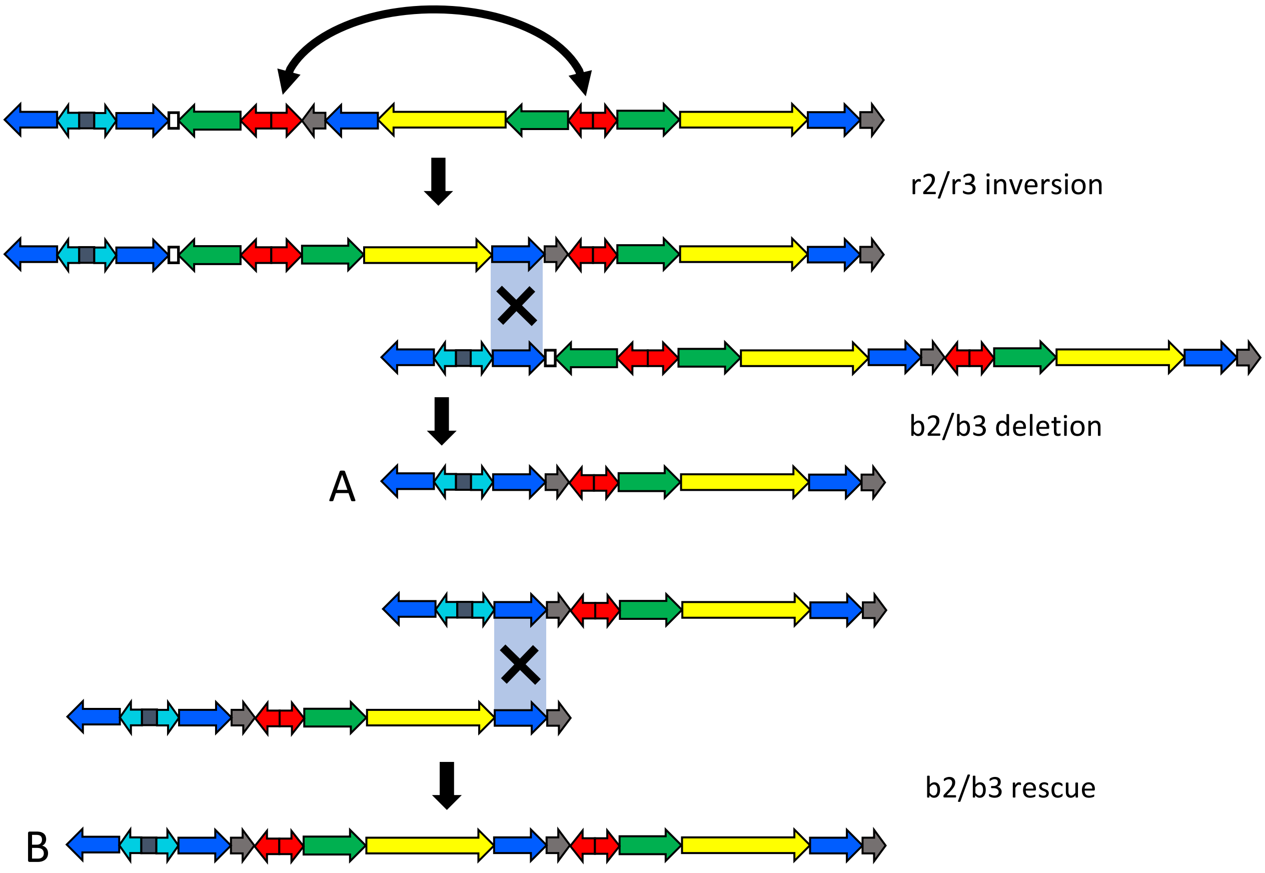

Supplementary figure 4. NAHR events between AZFc amplicons in Y haplogroup N. Structure A, found in the sample R063, is the result of a r2/r3 inversion followed by a b2/b3 deletion. A third event, a duplication that involve the resulting b2 and b3, lead to structure B, present in the samples R061, R064 and R065.

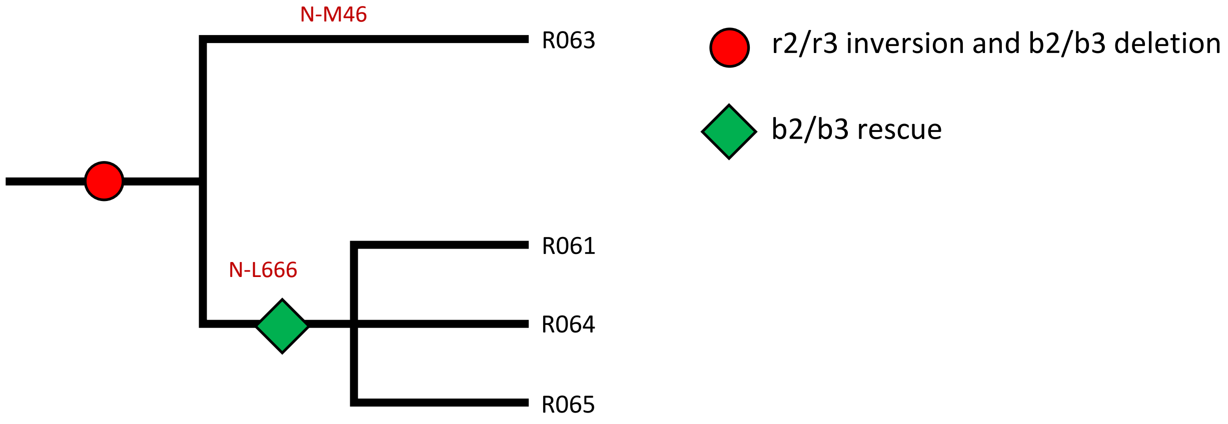

Supplementary figure 5. Schematic representation of the phylogenetic relationship among the N haplogroup samples here analyzed. The red dot and the green diamond indicate in which branch the NAHR events occurred.

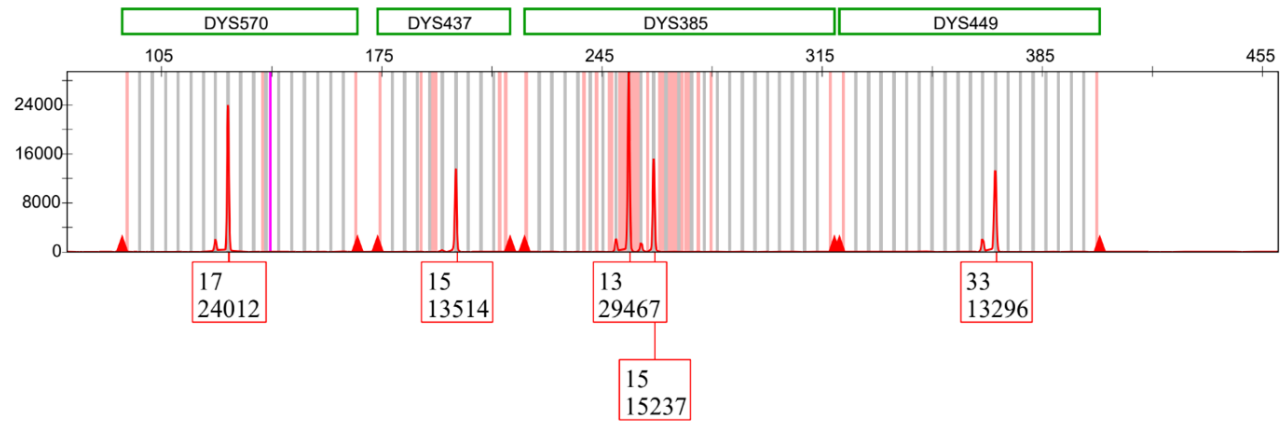

Supplementary figure 6. Portion of Yfiler Plus Y-STR profile showing a clear peak imbalance (2:1) at the DYS385 locus due to the duplication of one P4 arm.
